# Combined Antimicrobial Effect of Bio-Waste Olive Leaf Extract and Remote Cold Atmospheric Plasma Effluent

**DOI:** 10.3390/molecules26071890

**Published:** 2021-03-26

**Authors:** Jose Gustavo De la Ossa, Hani El Kadri, Jorge Gutierrez-Merino, Thomas Wantock, Thomas Harle, Maurizia Seggiani, Serena Danti, Rossella Di Stefano, Eirini Velliou

**Affiliations:** 1 Doctoral School in Life Sciences, University of Siena, 53100 Siena, Italy; josegustavo.delao@student.unisi.it; 2 Bioprocess and Biochemical Engineering Group (BioProChem), Department of Chemical and Process Engineering, University of Surrey, Guildford GU2 7XH, UK; h.elkadri@surrey.ac.uk; 3 Cardiovascular Research Laboratory, Department of Surgical, Medical and Molecular Pathology and Critical Care Medicine, University of Pisa, 56100 Pisa, Italy; rossella.distefano@unipi.it; 4 School of Biosciences and Medicine, University of Surrey, Guildford GU2 7XH, UK; j.gutierrez@surrey.ac.uk; 5 Fourth State Medicine Ltd., Longfield, Fernhurst, Haslemere GU27 3HA, UK; t.wantock@surrey.ac.uk (T.W.); t.harle@fourthstatemedicine.co.uk (T.H.); 6 Department of Civil and Industrial Engineering, University of Pisa, Largo L. Lazzarino 2, 56122 Pisa, Italy; maurizia.seggiani@unipi.it; 7 Interdepartmental Research Center “Nutraceuticals and Food for Health”, University of Pisa, 56100 Pisa, Italy; 8 Centre for 3D Models of Health and Disease, Division of Surgery and Interventional Science, University College London, London W1W 7TY, UK

**Keywords:** polyphenols, olive tree, green technology, antibacterial, food contamination, *S. aureus*, *E. coli*, *L. innocua*

## Abstract

A novel strategy involving Olive Leaf Extract (OLE) and Cold Atmospheric Plasma (CAP) was developed as a green antimicrobial treatment. Specifically, we reported a preliminary investigation on the combined use of OLE + CAP against three pathogens, chosen to represent medical and food industries (i.e., *E. coli*, *S. aureus* and *L. innocua*). The results indicated that a concentration of 100 mg/mL (total polyphenols) in OLE can exert an antimicrobial activity, but still insufficient for a total bacterial inactivation. By using plain OLE, we significantly reduced the growth of Gram positive *S. aureus* and *L. innocua*, but not Gram-negative *E. coli*. Instead, we demonstrated a remarkable decontamination effect of OLE + CAP in *E. coli*, *S. aureus* and *L. innocua* samples after 6 h. This effect was optimally maintained up to 24 h in *S. aureus* strain. *E. coli* and *L. innocua* grew again in 24 h. In the latter strain, OLE alone was most effective to significantly reduce bacterial growth. By further adjusting the parameters of OLE + CAP technology, e.g., OLE amount and CAP exposure, it could be possible to prolong the initial powerful decontamination over a longer time. Since OLE derives from a bio-waste and CAP is a non-thermal technology based on ionized air, we propose OLE + CAP as a potential green platform for bacterial decontamination. As a combination, OLE and CAP can lead to better antimicrobial activity than individually and may replace or complement conventional thermal procedures in food and biomedical industries.

## 1. Introduction

There is a growing interest in sustainable industrial routes, with special emphasis in the food and biomedical sectors, for the manufacture of safe yet sustainable packaging for edible products as well as surgical devices [1,2]. Consequently, the attention towards less impactful processing technologies capable of replacing conventional decontamination methods is increasing. To this end, natural antimicrobial compounds, including vegetable bioactive molecules, offer an emerging strategy to control microbial contamination. Indeed, some molecules and plant-derivatives demonstrate good antimicrobial activity against pathogenic bacteria found in packaging or processing phases in the food industry [1], as well as in biomedical devices, such as surgical tools and supporting equipment, the latter being very important in healthcare, since inaccurate sterilization is responsible for at least 1.5%–7.2% of post-operative complications [2]. The antibacterial properties of plants have been widely investigated [3]. In fact, a great variety of plant species containing components which exhibit antimicrobial activity against a wide range of Gram-positive and Gram-negative bacteria has been reported, including *Hibiscus*, *Rosmarinus officinalis*, *Thymus vulgaris*, *Malva sylvestris* and *Allium sativum*, among others [4,5,6,7,8,9,10,11,12,13,14]. Plant antimicrobials are more attractive than the synthetic food preservatives, as they are generally recognized as safe and capable of benefiting human health, including essential oils [15]. Olive Leaf Extract (OLE), an agricultural by-product obtained during the harvesting or pruning process of olive fruits, can be considered as a plant derivative entitled with both antimicrobial and antioxidant activities [16]. The leaves of olive trees (i.e., *Olea europaea*, *Oleaceae*) together with small and large branches produced from the cultivation and harvesting of olives represent waste biomasses, usually burned by farmers with consequent production of greenhouse gases [17]. As such, their conversion into higher-value products can represent a sustainable and eco-friendly alternative to their current disposal. To obtain OLE from olive oil leaves, a water-based extraction method can be applied in place of organic solvent solutions [18]. In fact, widely used OLE extraction procedures based on ethanol and methanol aqueous solutions have shown shortcomings such as low extraction efficiency, prolonged extraction times and high energy consumption for heating [19]. OLE is used in traditional medicine as a dietary supplement and an over-the-counter drug for a variety of beneficial effects on human health, such as lowering blood pressure and supporting the cardiovascular and immune systems [20]. OLE and the other parts of the olive tree contain considerable amounts of polyphenols [18]. Specifically, OLE contains oleuropein, the main phenolic compound found in unprocessed olive fruits and leaves, up to 140 mg/g and 60–90 mg/g, respectively [21]. Oleuropein is a glycosylated seco-iridoid glucoside composed of elenolic acid and hydroxy-tyrosol; it has an oleosidic skeleton that is common within the seco-iridoid glucosides of Oleaceae, mainly in its aglycone form, which makes the sugar moiety insoluble in oil [22].According to some studies, OLE exerts antimicrobial effects due to its high phenolic content [16,23,24]. They report the growth inhibition in some bacteria species, reported in Table 1. However, these findings are contradictory and may depend on the concentration and/or the bacteria used. For example, Sudjana et al. showed that OLE had appreciable antimicrobial activity only against *C. jejuni*, *H. pylori* and *Staphylococcus* spp., but poor effect against *B. subtilis*, *Candida* spp., *E. coli*, *K. pneumoniae*, *P. aeruginosa* and *S. marcescens* [25]. Djanane et al. showed that 5% OLE was more efficient as compared to 1% OLE against the pathogens *Salmonella* and *E. coli* O157:H7 in Halal meat [26]. Albertos et al. reported that edible films with OLE at 5.6% (*w*/*w*) reduced *L. monocytogenes* growth on smoked salmon [27]. Furthermore, in a study by Pereira et al., OLE was screened for its antimicrobial activity against *B. cereus*, *B. subtilis*, *S. aureus*, *E. coli*, *P. aeruginosa*, *K. pneumoniae* bacteria and against the fungi *C. albicans* and *C. neoformans*, showing *B. cereus* and *C. albicans* to be the most sensitive to OLE [28]. Therefore, the use of OLE as antimicrobial treatment is still a matter of debate due to the contradictory results of the insufficient number of systematic studies conducted so far [29,30].

Another emerging non-thermal technology with potential applications in several different industries, including safe and sustainable food production, is Cold Atmospheric Plasma (CAP). Plasma is commonly referred to as the fourth state of matter, namely, an ionized state of gas which exhibits unique properties. The plasma state is pervasive, being found in diverse entities (e.g., stars, interstellar space, lighting technology). Cold plasma (CP) is commonly obtained by application of a strong electromagnetic field to a neutral gas that induces ionization. CP is composed of ions, electrons, free radicals, excited atoms/molecules and photons of various wavelengths. CAP treatment in the presence of air generates several reactive short and long-lived species including reactive oxygen species (ROS) and reactive nitrogen species (RNS), which have been shown to play a dominant role in antibacterial and biological activity [31,32,33]. CAP, due its non-thermal nature, is a potential alternative to conventional methods such as the use of chlorine and thermal processes (i.e., drying, chilling, freezing and pasteurization) to improve microbiological safety in food packaging [34].

In the biomedical field, this technique can be applied in surface disinfection, sterilization of surgical instruments and decontamination of devices [2]. Moreover, plasma devices, such as indirect argon plasma (e.g., MicroPlaSter alpha and beta), have been applied in a clinical trial in patients with chronic infected wounds [2]. Some studies reported that CAP exhibited excellent antibacterial efficacy against target food pathogens, their spores and biofilms [35]. In addition, new information has been elucidated explaining the effective application of CP in functional packaging, for elimination of toxins and degradation of pesticides [36]. Plasma has also been investigated as a pre-treatment step to activate or modify material surfaces, since it can improve the efficiency of post-grafting or the incorporation of antimicrobial components onto the surface [37]. Chang et al. used plasma pre-treatment to promote the grafting of chitosan on polyester fabrics to obtain antibacterial activity against *B. subtilis* and *S. aureus* [37]. In this study, fabrics were previously pre-treated by an argon/oxygen (Ar/O_2_) dielectric barrier discharge (DBD) plasma for surface activation, subsequently exposed to the atmosphere for further oxidization and, finally, immersed in a chitosan solution for chitosan grafting. Other natural compounds, such as nisin peptides, thymol and herbs, have also been grafted onto plasma pre-treated polymer surfaces to obtain an antibacterial material [38,39,40]. Different types of plasma pre-treatments, namely N_2_ and Ar/O_2_ plasma modifications, and plasma-induced grafting of acrylic acid have been used to incorporate nisin peptides onto the surface of low density poly-ethylene films [40]. Other interesting developments using whey protein formulations as coating strategies on polyethylene terephthalate films, pre-treated by corona discharge of CAP, resulted in excellent barrier properties, making the packaging efficacy comparable to the ethylene vinyl alcohol copolymers barrier layer, conventionally used in food packaging composites [41]. Similarly, in another study, the application of plasticized corn–zein coating on corona-discharge-treated polypropylene films for the flexible packaging industry showed more than three orders of reduction in oxygen permeability [42]. Hence, although the antimicrobial properties of plain OLE is still debated, OLE can be used in combination with CAP treatment (OLE + CAP) to empower antimicrobial activity against bacteria present in the food and biomedical industries. In this way, it is expected to replace or complement conventional sterilization and decontamination processes that use high energy with those of lower environmental impact [43,44]. Overall, both OLE and CAP are sustainable technologies for bacterial decontamination purposes; however, singularly, their action is not as strong and efficient as heat sterilization. The OLE + CAP combination represents a novel approach that involves the bioactive molecules of OLE and ROS/RNS generated in situ by CAP. In this study, the antibacterial activity of OLE, CAP and OLE + CAP against common bacterial species affecting both food and medical devices was preliminarily investigated to determine potential synergistic effects between the two approaches. Having a combined green technology for bacterial decontamination would allow more sustainable and better management of human health.

## 2. Results

### 2.1. Effect of OLE on Bacterial Strains

**Figure 1:**
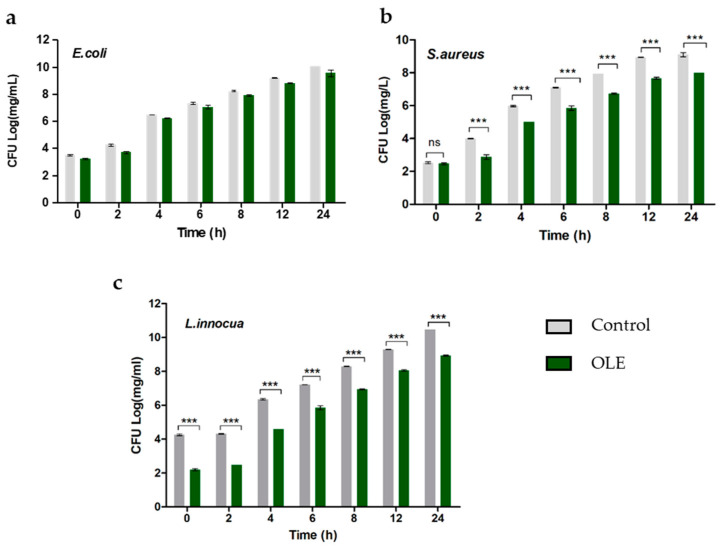
Bar graphs showing the effect of OLE administration at 100 mg/mL total polyphenols (TPs) on the growth of different bacterial strains: (**a**) *E. coli*, (**b**) *S. aureus*, (**c**) *L. innocua*, up to 24 h. The values are reported as mean ± standard error of the mean (SEM) (n = 3), significance at *p* < 0.05 by One-way ANOVA and Tukey′s HSD post hoc test; *** *p* < 0.0001, n.s. not significant.

The OLE used was extracted from *Olea europaea* var. *Olivastra seggianese* in Tuscan cultivar [45]. The antibacterial activity was evaluated in vitro against gram-negative *E. coli* and gram-positive *S. aureus* and *L. innocua* strains. Each resuspended *E. coli*, *S. aureus* and *L. innocua* sample was separately treated with OLE at 100 mg/mL total polyphenols (TPs), as this concentration is within the range of efficiency [46]. The effect of OLE on the different bacteria is illustrated in Figure 1. OLE composition is given in Table 2.

For *E. coli*, OLE did not exhibit any significant antimicrobial effect (Figure 1a). Conversely, in *S. aureus* and *L. innocua* statistically significant antimicrobial effects were observed over time (Figure 1b,c). In particular, for *L. innocua* OLE showed a reduction in the number of colonies even if inactivation was not reached (Figure 1c).

### 2.2. Effect of OLE + CAP on Bacterial Strains

**Figure 2:**
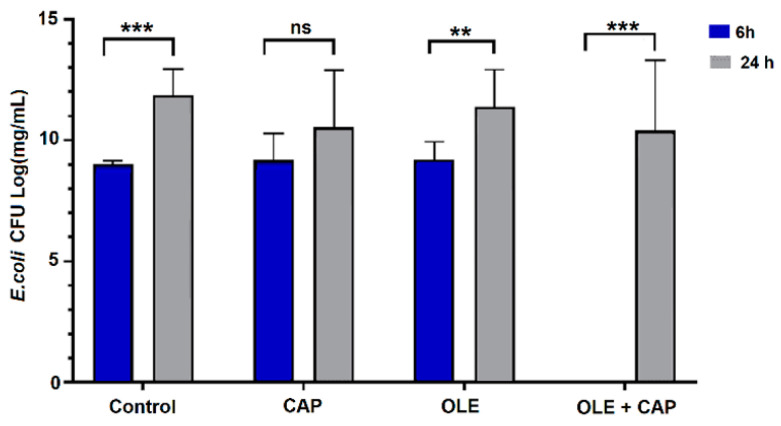
The effects of Cold Atmospheric Plasma (CAP) (1 min exposure time) and/or OLE on *E. coli*. The values are reported as mean ± standard error of the mean (SEM) (n = 3), significance at *p* < 0.05 by One-way ANOVA and Tukey′s HSD post hoc test; ** *p* < 0.001, *** *p* < 0.0001, n.s. not significant.

**Figure 3:**
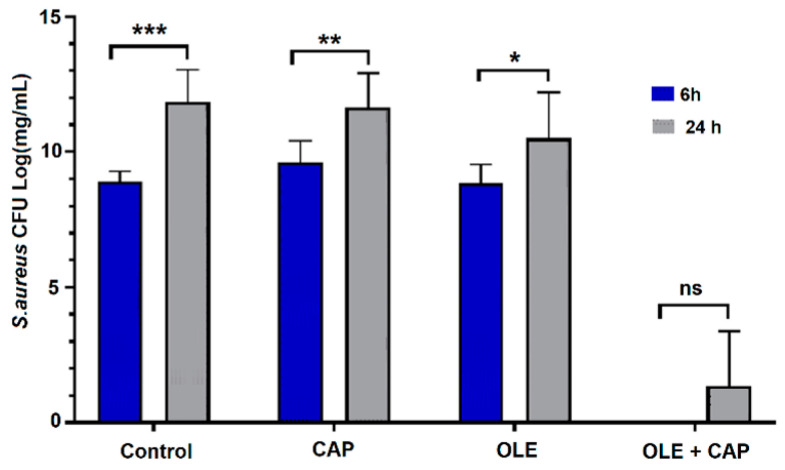
The effects of CAP (1 min exposure time) and/or OLE on *S. aureus*. The values are reported as mean ± standard error of the mean (SEM) (n = 3), significance at *p* < 0.05 by One-way ANOVA and Tukey′s HSD post hoc test; * *p* < 0.01, ** *p* < 0.001, *** *p* < 0.0001, n.s. not significant.

**Figure 4:**
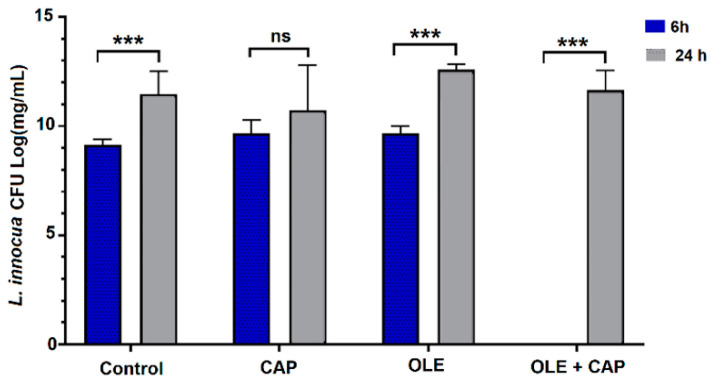
The effects of CAP (1 min exposure time) and/or OLE on *L. innocua*. The values are reported as mean ± standard error of the mean (SEM) (n = 3), significance at *p* < 0.05 by One-way ANOVA and Tukey’s HSD post hoc test; *** *p* < 0.0001, n.s. not significant.

The potential synergistic effect of OLE and CAP was evaluated at two relevant time points, 6 h and 24 h after application of OLE, CAP or OLE + CAP, using previously published methods [47,48,49]. The comparison of results obtained after 6 h and 24 h using the single treatment and their combination is reported in Figure 2, Figure 3 and Figure 4. Using the combined OLE + CAP treatment, *E. coli* had significant inhibition at 6 h, while at 24 h the bacteria reproduced, but still with a significant reduction with respect to the control and individual treatments (Figure 2). The most remarkable effect was obtained against *S. aureus* (Figure 3).

*S. aureus* samples subjected to OLE + CAP treatment significantly decreased after 6 h and 24 h compared to control and single treatments. The effectiveness of the combined treatment was confirmed by a very low replication of residual bacteria at 24 h.

The case of *L. innocua* was more complex. After a high eradication, at 6 h the bacteria started reproducing and grew up to control levels (Figure 4).

For this strain, the plain OLE was capable of controlling bacterial growth over time. In *L. innocua*, bacterial decontamination was observed after 6 h, but the effect was recovered after 24 h, reaching ~7∙Log [CFU]. Interestingly, and in accordance with the results reported in Figure 1, OLE alone demonstrated improved effectiveness in containing *L. innocua* growth.

Overall, the combined treatment (OLE + CAP) showed a synergistic antimicrobial effect against *E. coli*, *S. aureus* and *L. innocua* at 6 h. However, the remaining bacteria in small numbers were able to grow over longer time. The combined OLE + CAP treatment allowed a remarkable bacterial inactivation in terms of colony forming units (CFUs) during the exponential phase (6 h) for all the strains. In the stationary phase (24 h), the OLE + CAP treatment was sufficiently effective on *E. coli*, which grew by reaching ~7∙Log [CFU] and was still remarkably effective on *S. aureus*, which grew up to ~3∙Log [CFU], thus confirming the overall synergistic effect of OLE + CAP treatment. Differently, the individual treatments did not demonstrate relevant decontamination activity under our experimental conditions.

## 3. Discussion

The search for novel technologies for bacterial decontamination is a topic of intense research, which encompasses the use of chemical, thermal, radiation and combined treatments. A number of sectors can benefit from safe and green methods, such as bactericidal agents, in particular the food and health industries [1,2,3]. In time, microbes have started to become resistant to many decontamination technologies used on a large scale, thus leading to the need for newer and more powerful antibacterial agents. On the other hand, health and environmental factors are driving the industry towards less aggressive and better sustainable methods to treat their products [17,18,19,34]. However, low energy approaches are often less effective than their conventional counterparts. As several plants are known to possess antimicrobial properties, new research is also focusing on analyzing and using plant extracts with antibacterial purposes along with combined treatments, for application on an industrial scale [15,40]. The research on this topic is still fragmented, incomplete and controversial, due to the fact that biological derivatives, including OLE, greatly vary in their outcomes [29,30]. As such, better detailed and more systematic investigations are necessary to combine plant derivatives with other technologies to enhance antimicrobic efficacy.

We reported a preliminary investigation on the combined use of OLE + CAP against three pathogens, chosen to represent medical and food industries (i.e., *E. coli*, *S. aureus* and *L. innocua*). Since OLE derives from a bio-waste and CAP is a non-thermal technology based on ionized air, we propose OLE + CAP as a novel green approach potentially useful for bacterial decontamination.

It important to consider that OLE has a high variability in the concentration of bioactive compounds, as a consequence of various factors, such as the raw material and the extraction process, among others [50]. Moreover, the final composition of the OLE has great importance for its antimicrobial efficacy. Furthermore, broad-spectrum polyphenols have been found in OLE at small concentrations (Table 2), which can probably exert an antimicrobial effect together with the most abundant compound, oleuropein. In our study based on the surface spread method, the bacterial inactivation of plain OLE was evident (*p* < 0.0001) in *S. aureus* and *L. innocua* at all the time points up to 24 h, while *E. coli* did not show a considerable susceptibility to OLE. As a gram-negative bacterium, *E. coli* is in fact more resistant to conventional methods regarding its eradication [27]. The obtained results indicated that the presence of several phenolic compounds in 100 mg/mL TPs can exert an antimicrobial activity, but still insufficient to obtain a total or significant bacterial inactivation. It is reported that the polyphenols in OLE, or possibly the synergistic effects among them [16], may be responsible for OLE antimicrobial activity by inducing membrane permeability in bacteria, further inhibition of biochemical pathways and, finally, disintegration of the outer membrane leading to bacterial cell death [51].

The CAP effluent works at room temperature (RT) and atmospheric pressure, which eliminates the need for expensive noble gases, making it economically feasible on an industrial scale. CAP application leads to chemical species, such as ROS/RNS, which in combination with the polyphenolic compounds of OLE, mainly oleuropein, are supposed to inactivate the microorganisms. Recent investigations have shown that CAP efficacy is directly correlated to bacterial cell wall thickness in several species [52,53]. Gram-negative species, such as *P. aeruginosa*, were almost completely eradicated due to their thin cell membrane (2.4 nm cell wall), while Gram-positive species, such as *B. subtilis*, displayed the highest resistance to CAP, possessing thicker membranes (e.g., 55.4 nm cell wall). *E. coli* have a thinner outer membrane compared to the Gram-positive *S. aureus* and *L. innocua*. However, no clear trend is apparent from this and other studies, since complex interactions with the system, process, surface or medium may also impact on CAP efficacy in combination with cell type.

Another significant role in the mechanical disruption of the bacterial cell membrane is the effect of charged particles that could accumulate on the surface and cause electrostatic stress [53]. The reactive species produced in plasma react with the protein amino-acids and cause further structural changes in proteins, finally destroying the quiescent cells [54]. It is hypothesized that such morphological changes overcome the tensile strength of the cell membrane [55]. In fact, *S. aureus* demonstrated a size reduction of colonies (results not shown). Cell membrane perforation induced by etching enhances the diffusion of secondary reactive species that might be formed in the plasma discharge inside the cell [31]. CP, due to its complex composition and multiple different reactive components, is expected to play a role, independently or synergistically, in the inactivation of microbial targets. Generally, the efficacy of CP depends on the device design and system operating parameters, such as gas composition, flow rate, moisture, temperature, voltage, and frequency [31,50,56]. In addition, the ozone O_3_, present in CAP effluent could break structural bonds in the peptidoglycan component of the cell wall, such as C–O, C–N bonds, leading to cell wall destruction and, consequently, cell death [56,57].

On the basis of the results obtained, it can be deduced that CAP, containing ROS/RNS [58], combined with the action of the OLE polyphenols, exerts an enhanced antimicrobial activity by efficient damage and disruption of the bacterial membrane [52,53]. In fact, in combination with OLE, a better inactivation was also obtained with *S. aureus*, a Gram-positive bacterium, probably due to ROS-enhanced intracellular damage [53]. Efficacy of the combined effect of CAP and nisin against *L. innocua*, grown planktonically or as surface colonies in a food model, has recently been reported as another application for bacteria eradication [59], which is suggestive of a potentiated effect of CAP in combination with selected biomolecules. In fact, in our findings, OLE alone demonstrated improved effectiveness in containing *L. innocua* growth with respect to OLE + CAP, in which, after a first significant decrease, the bacteria grew again to control level. Especially in this case, higher time exposure to CAP or more concentrated OLE could be needed to show a more prolonged effect of bacterial eradication.

All in all, we demonstrated the remarkable effect of OLE + CAP in sample decontamination by *E. coli*, *S. aureus* and *L. innocua* after 6 h. This effect was best maintained up to 24 h using the *S. aureus* strain. On the other hand, *E. coli* and *L. innocua* grew again after 24 h. In the latter case, OLE alone was most effective by significantly reducing bacterial growth. As the most innovative approaches also consider developing material surfaces with intrinsic antimicrobial properties, e.g., by virtue of nanostructures inhibiting bacterial growth and biofilm formation [60,61], the addition of OLE to those surfaces followed by CAP effluent application could provide a robust antimicrobial strategy. As a combination, OLE and CAP can lead to better antimicrobial activity than individually and may replace or complement conventional thermal procedures in the food and biomedical industries. However, a multifactorial study that takes into account the type of bacteria, time and mode of exposure to CAP, content and type of polyphenols of OLE and overall cost-effectiveness, safety and sustainability is needed to optimize the process for industrial use. For enhanced safety, innovative intelligent labels could be applied to the OLE + CAP packaging to properly monitor the sterilization process as well as the storage conditions [62].

The availability of effective and low cost green technologies to disinfect edible and medical products would greatly impact the management of food- and healthcare-associated infections, overall estimated to affect 30 million people annually in Europe.

## 4. Materials and Methods

### 4.1. OLE Extraction and Characterization

OLE was extracted from *Olea europaea* var. *Olivastra seggianese* cultivar. The collection of the leaves from which OLE was extracted was performed at CNR-IVALSA, Follonica (GR), Italy. TP content was determined according to the Folin-Ciocalteu method using gallic acid as the standard equivalent (µg GAE/mg), purchased from Merk (Darmstadt, Germany) [45]. A high-performance liquid chromatography analysis (HPLC) was carried out to identify and quantify the major phenolic compounds of the obtained OLE.

### 4.2. CAP Technology

A dielectric barrier discharge reactor was used for CAP inactivation provided by Fourth State Medicine Ltd. To generate CP, the instrument was set up at a flow rate of 5 L/min air for 1 min. The samples were treated at RT (approx. 20 °C). The plasma power supply was set at 8 kV voltage and 20 kHz AC frequency.

### 4.3. In Vitro Tests

Inoculum was prepared from stock cultures of *E. coli* (ATCC 47076), *S. aureus* (ATCC 25923) and *L. innocua* (ATCC 33090), previously stored at −80 °C in Tryptic Soy Broth (TSB, Oxoid Ltd., UK), supplemented with 15% v/v glycerol (Merk, Darmstadt, Germany). More specifically, a loopful of thawed stock culture was inoculated in 15 mL TSB for 24 h at 37 °C. Subsequently, 20 μL was transferred to fresh 20 mL TSB and cultured at 37 °C for either 6 h or 24 h to obtain bacterial cells in the exponential or stationary phase, respectively, following a procedure reported in previous studies [48,49,50]. Thereafter, 1 mL taken from the 6 h and 24 h cultures was centrifuged at 10,000× g for 10 min at 23 °C and re-suspended in 1 mL of phosphate saline buffer (PBS; Merk). Each resuspended bacteria sample was separately treated with OLE at 100 mg/mL TPs, as it is higher than other concentrations demonstrating antibacterial activity as previously tested [45]. In a separate 24-well plate, 900 mL of treatment solution (composed by OLE dissolved in TBS) with 100 mg/mL TPs was added to 100 µL of bacterial inoculum. The effects of the OLE on the strains were evaluated at different times for a total duration of 24 h by plate counting (CFU/mL). The potential synergistic effect of OLE and CAP was evaluated at two relevant time points, 6 h and 24 h after application of OLE, CAP or OLE + CAP. The samples to be treated with CAP were exposed soon after OLE addition, for 1 min using air (5.0 L/min) at atmospheric pressure and RT. All experiments were carried out in triplicate to ensure statistical significance.

### 4.4. Statistical Analysis

Statistical analysis was performed using SPSS 22.0 software (SPSS Inc., Chicago, IL, USA) by conducting one-way analysis of variance (ANOVA, San Francisco, CA, USA) and the Tukey′s honestly significant difference (HSD) post hoc test to determine any statistically significant difference among samples. Significance was set at *p* < 0.05.

## 5. Conclusions

In this study, the antimicrobial effects of CAP, OLE and their combination against bacterial pathogens, i.e., *E. coli*, *S. aureus* and *L. Innocua*, was investigated. The combined OLE + CAP treatment had substantial antimicrobial activity against the bacterial species under study at early time points (6 h), whereas individual CAP and OLE treatments showed comparatively poor effects under the conditions surveyed. At the conclusion of this preliminary study on the combined antimicrobial effect of CAP and OLE, a synergistic effect during the exponential phase was evident, suggesting that the combination of these sustainable approaches could offer an innovative strategy for providing microbiological safety in the biomedical and food industry. We can hypothesize the use of OLE as a coating or polymer blend in food or medical packaging, followed by CAP treatment, to ensure a green and safely decontaminated environment.

## Figures and Tables

**Table 1:** Bacterial growth inhibition studies with olive leaf extract (OLE) treatment on bacteria strains.

Bacterial Species	Olive Variety and Origin	OLE Extraction Method	OLE Concentration	Reference
*K. pneumoniae*	*Olea europaea* (Turkey, west Anatolian)	Aqueous	30 µL OLE; 15% (*w*/*v*)	[22,30]
*S. aureus*	*Olea europaea* (Portugal)	Aqueous	5 mg/mL	[28,29]
*B. cereus*	*Olea europaea* (Portugal)	Aqueous	5 mg/mL	[28]
*B. subtilis*	*Olea europaea*	Ethanol	27.2 ± 0.99 mg/g	[28]
*P. aeruginosa*	*Olea europaea* (Portugal)	Aqueous	5 mg/mL	[28]
*C. jejuni*	*Olea europaea* (Australia)	n.a.	n.a.	[27]
*H. pylori*	*Olea europaea* (Australia)	n.a.	n.a.	[27]
*E. coli*	*Olea europaea* (Several countries)	Water, ethanol.	Variable	[28,30,31]
*S. enterica*	*Olea europaea* subsp. *europaea var. Sylvestris* (Algeria)	Methanol/water	198.7 ± 3.6 mg GAE/g	[27,28,30]
*L. monocytogenes*	Commercial extract (USA)	Water/ethanol	62.5 mg/mL	[27,30]

**Table 2:** Concentration of the main polyphenols in OLE.

OLE Composition	Concentration (mg/g OLE)
Oleuropein	32.64 ± 3.06
Luteolin-7-O-glucoside	6.97 ± 0.24
Rutin	3.37 ± 0.33
Apigenin-7-O-glucoside	1.97 ± 0.17
Hydroxy-tyrosol	0.85 ± 0.08
Caffeic acid	0.18 ± 0.02

## Data Availability

Not applicable.

## Sample Availability

Samples of OLE are available from the authors (J.G.D.l.O.).
